# Associations between Experiences of Police Contact and Discrimination by the Police and Courts and Health Outcomes in a Representative Sample of Adults in New York City

**DOI:** 10.1007/s11524-021-00583-6

**Published:** 2021-11-22

**Authors:** Azure Thompson, María Baquero, Devin English, Michele Calvo, Simone Martin-Howard, Tawandra Rowell-Cunsolo, Marné Garretson, Diksha Brahmbhatt

**Affiliations:** 1 SUNY Downstate School of Public Health, Brooklyn, NY USA; 2 NYC Department of Health and Mental Hygiene, Queens, NY USA; 3 Rutgers University, New Brunswick, NJ USA; 4 New York Academy of Medicine, New York, NY USA; 5 Long Island University-Brooklyn, Brooklyn, NY USA; 6 University of Wisconsin, Madison, WI USA; 7 Weill-Cornell Medical College, New York, NY USA

**Keywords:** Social environment, Social marginalization, Social discrimination, Mental health, Physical health, Binge drinking, Stress

## Abstract

Communities marginalized because of racism, heterosexism, and other systems of oppression have a history of being aggressively policed, and in those contexts, researchers have observed associations between a range of negative experiences with police and poor physical, mental, and behavioral health outcomes. However, past studies have been limited in that experiences of police contacts were aggregated at the neighborhood level and, if police contacts were self-reported, the sample was not representative. To address these limitations, we employed NYC Department of Health and Mental Hygiene 2017 Social Determinants of Health Survey (*n* = 2335) data to examine the associations of self-reported police contacts and discrimination by police and the courts with measures of physical (poor physical health), mental (poor mental health, serious psychological distress), and behavioral health (binge drinking). Residents marginalized because of racial, ethnic, and sexual minority status were more likely to be stopped, searched, or questioned by the police; threatened or abused by the police; and discriminated against by the police or in the courts; those experiences were associated with poor physical, mental, and behavioral health outcomes. The associations between experiences with police and poor health outcomes were strongest among Black residents and residents aged 25–44. Our findings suggest that the health of NYC residents who have had exposure to police and experienced discrimination by the police and courts is poorer than those who have not, and build on a growing body of evidence that aggressive policing practices have implications for public health.

## Introduction

Communities marginalized because of racism, heterosexism, and other systems of oppression in New York City (NYC), and other large urban areas in the United States (US), have experienced a long history of aggressive policing [1–3]. Aggressive policing is a practice in which pedestrians are stopped for low-level offenses or suspicion of an offense with the intention of preventing more serious crime [3]. The conceptual origin of aggressive policing practices is the Broken Windows Theory developed by social scientists George Kelling and James Wilson and introduced in *The Atlantic* magazine in 1982 [4]. The untested yet widely accepted theory hypothesized that signs of the disorder, such as a broken window, are a signal of limited neighborhood collective efficacy and social cohesion and control, and consequently, there is no formal or informal punishment in the neighborhood for acts of disorder. Thus, reducing serious crime starts with the prevention of minor offenses that create the appearance of disorder. When applied in practice, this theory shifts policing focus to low-level crimes [4]. Quality-of-life (QOL) policing, introduced in NYC in 1994, was an early iteration of an aggressive policing policy. QOL policing targeted low-level, nonviolent offenses for which a person could be arrested or issued a summons [5], both requiring adjudication in the court. Another aggressive policing practice is stop, question and frisk (SQF). Introduced in 2003, SQF permitted police officers to stop and question a person based on probable cause, then frisk the person for weapons or other contraband [1]. In 2013, this practice in NYC was found by a federal judge to be unconstitutional and racially discriminatory [6].

Aggressive policing practices operate at multiple levels to compound marginalization of communities already marginalized because of racism [7] and heterosexism [8]. At the institutional level, aggressive policing practices such as SQF are concentrated among socially disadvantaged communities [9]. For example, Black and Latino New Yorkers are disproportionately stopped and ordered to appear in court, with nearly 12,000 Black and Latino New Yorkers stopped by the police in 2019, compared to just over 1000 White New Yorkers [10]. The disparity at the intersection of age, race, and gender is more stark. For example, the average yearly rate of stops between 2004 and 2012 was highest among 18-year-old Black male New Yorkers at 976 per 1000 Black males. The rate of stops for 18-year-old White male New Yorkers was well below 250 per 1000 White males [11]. At the interpersonal level, Black, Latino, and sexual minority individuals have been found across a number of studies to be more likely to report experiencing discrimination—differential treatment based on social identity—when interacting with the police than individuals not occupying those identities [12–17]. Another study found that among individuals with recent court experiences, Black individuals were more likely than White individuals to report having negative experiences, including perceiving court procedures and outcomes as unfair and low levels of concern and respect displayed by the court [18].

As a consequence of differential practices by the criminal legal system based on race, ethnicity, and sexual identity, socially marginalized communities might bear a disproportionate burden of poor health from aggressive policing. The ecosocial theory posits that it is the embodiment of socially structured—patterns of human relationships by social position—conditions that contribute to the distribution of disease in a population [19]. Populations positioned lower on the social structure, or socially marginalized populations, because of racism, heterosexism, or other systems of oppression are differentially exposed—contemporarily and historically—to negative conditions that render them vulnerable to disease via diverse, concurrent, and interacting pathways that accumulate over the life course [19]. Aggressive policing practices are socially structured exposures [7, 8] that can increase vulnerability to disease via stress pathways [20] linked to mental [20] and physical [21] health outcomes. In addition, these practices can also increase the risk of poor behavioral health outcomes such as excessive alcohol use to cope with the stress of a police-initiated contact [22, 23]. Past analyses indicate that police stops and abuse are associated with poor mental, physical, and behavioral health outcomes [24]. Studies have also found discrimination by the police is associated with health; if an individual experienced a police encounter as discriminatory, the association with poor mental health was stronger [25].

Previous studies on police encounters and health outcomes, however, have had several limitations. Prior studies that used representative samples either did not take into account factors known to be associated with health outcomes (e.g., education and income) [24] or solely employed an aggregated neighborhood-level police exposure [26]. To address these gaps, we examine the associations of self-reported police stops and abuse and discrimination by the police and courts, with poor physical, mental, and behavioral health outcomes in a representative sample of adult residents in NYC. Our hypotheses are informed by the ecosocial theory. We hypothesize that there will be a positive association between police stops, abuse by the police, and discrimination by the police and courts and poor health outcomes. Considering the embodiment of socially structured conditions [19], we also examine whether these associations vary by race and ethnicity. Moreover, the embodiment of socially structured conditions [19] accumulates over the life course [19]; therefore, we also examine whether these associations vary by age. We hypothesize that the associations will be stronger among individuals who experience racism, such as Black and Latino populations, and among those who are older.

## Methods

### Data

We analyzed data from the 2017 Social Determinants of Health (SDH) Survey, a cross-sectional survey of 2335 adults aged 18 and older conducted by the NYC Department of Health and Mental Hygiene to assess health and wellness. The SDH survey sample was drawn using both random digit dialing and address-based sampling, with surveys completed via landline telephone or cell phone interviews (*n* = 1433), web (*n* = 247), or pencil-and-paper (*n* = 655). With the purpose of having data be representative of the NYC adult population, data collected from telephone interviews were adjusted for initial probability of selection, dual cell phone and landline use, and non-response. Data collected from landline interviews were additionally adjusted for the probability of respondent selection in a household with multiple adults and for respondents in a household with two or more landlines. The survey had an overall response rate of 11.6% and cooperation rate of 80.4%, using modified versions of the American Association for Public Opinion Research’s (AAPOR) Response and Cooperation Rates #3 [27].

Estimates presented here are based on self-reported data, which were weighted to represent the 2015 NYC adult residential population (*n* = 6,585,635) using American Community Survey demographic data. The weighting approach reduced differences in health measures across survey modes [28]. Data were age adjusted to the United States 2000 standard population. Phone interviews were conducted in English, Spanish, Russian, Mandarin, and Cantonese; surveys completed online and by pencil-and-paper were in English.

### Measures

We focused on three exposures of interest: self-reported lifetime experience of being stopped by police, assessed with the question “Have you ever been stopped, searched, or questioned by the police?;” self-reported lifetime experience of threats or abuse by the police, assessed with the question “Have you ever been physically threatened or abused by the police?;” and lifetime experience of racial discrimination by the police or court system, assessed with the question “Have you ever experienced discrimination, been prevented from doing something or been hassled or made to feel inferior because of your race, ethnicity, or color from the police or in the courts?” Below we refer to each of these constructs as being stopped by the police, experiencing police abuse, and experiencing discrimination by police or in courts, respectively.

We examined four physical, mental, and behavioral health outcomes. In alignment with the measures used by the Behavioral Risk Factor Surveillance System (BRFSS), poor physical health and mental health were defined by the reported number of days that each was not good in the last 30 days, dichotomized as poor (14 days or more) versus not poor (13 days or less) for both physical and mental health [29, 30]. Also in line with BRFSS measurements [29], serious psychological distress in the last 30 days was defined by a score of 13 or greater on the Kessler Psychological Distress Scale (K6), based on analyses to determine the optimal cut point for classification accuracy [31]. We used the definition of the National Institute of Alcohol Abuse and Alcoholism to examine binge drinking, defined as having 5 or more drinks for men and 4 or more drinks for women on a single occasion in the last 30 days [32].

### Analysis

We calculated age-adjusted prevalence estimates of being stopped by the police, abused by the police, or experiencing discrimination by police or in courts by sociodemographic characteristics and health outcomes and behaviors (PROC DESCRIPT [33]; we used CONTRAST [33] statements to perform 2-tailed tests of significance (*α* = 0.05)).

We first evaluated the association between each of the exposures of interest and the selected health outcomes using multivariable logistic regression models (PROC RLOGIST and PRED_EFF [33] for pairwise comparisons). As most outcomes were relatively common (prevalence > 10%), we used the ADJRR [33] statement to obtain model-adjusted risk ratios (RRs) and 95% confidence intervals (CIs); this approach computes the ratio of predicted marginal proportions for logistic regression models. Multivariable models were adjusted for potential confounders of the relationship between our exposures and outcomes of interest, based on our review of extant literature [24–26]: nativity status (born in the US or elsewhere), household income (< 200% versus ≥ 200% of the Federal Poverty Level, FPL), and education (less than high school, high school graduate, some college, and college graduate). We investigated the potential collinearity of variables in the models and found variation inflation factors did not exceed 2.22, indicating low collinearity.

Based on our interest in examining differences across socially structured groups and the accumulation of negative conditions experienced by populations low on the social structure, we then explored effect measure modification to determine whether the strength of the association varied by race and ethnicity (Black, Latino, White, Asian/Pacific Islander, Other/Multi-race), by age (18–24, 25–44, 45–64, 65 +), and by sex (male/female) by testing 2-way interaction terms (*α* = 0.1). Statistical analyses were performed using SAS Enterprise Guide 7.15 software (Cary, NC) and SAS-callable SUDAAN 11.01 software (Research Triangle Institute, Research Triangle Park, NC) to account for the complex survey design. Statistical significance was determined at two-sided *p*-values < 0.05.

## Results

Overall, 29.3% of NYC adult residents reported having ever been stopped by police (weighted *N* = 1,941,000), 8.7% reported having ever experienced police abuse (weighted *N* = 577,000), and 14.6% reported having ever experienced discrimination by police or in courts (weighted *N* = 950,000). Those who reported having ever been stopped by police were more likely to be male; be gay, lesbian or bisexual; be born in the US; have a household income equal to or greater than 200% of the Federal Poverty Level (FPL); and not be in the labor force (Table 1). They were less likely to be Asian/Pacific Islander and to be aged 65 years and older. People who reported having ever experienced police abuse were more likely to be male; be Black or Other/Multi-race; be born in the US; have a household income less than 200% of FPL; and have completed some college, completed high school, or have education less than a high school degree. They were less likely to be older than 65 years of age. People having experienced discrimination by police or in courts were more likely to be male; be Black or Latino or Other/Multi-race; be born in the US; have a household income less than 200% of FPL; and have a high school degree or less than a high school education. NYC residents who reported having ever been stopped by police or having ever been abused by police were more likely to report poor physical health in the past 30 days (17.5% and 29.4%, respectively), as compared to those who had not had these experiences (11.0% and 11.8%, respectively) (Table 2). People reporting having ever been stopped by police, having ever been abused by police, or having ever experienced discrimination by police or in courts were approximately twice as likely to report poor mental health in the last 30 days (20.4%, 26.7%, and 21.3%, respectively) compared to those who had not had these experiences (11.9%, 13.5%, and 13.3%, respectively). Similarly, people who had ever experienced police abuse were over twice as likely (13.8%) as those who had not (5.8%) to report serious psychological distress, and over one-quarter of people ever stopped by police reported binge drinking (27.4%), compared to 17.9% of those never stopped by police.

**Table 1:** Prevalence of ever being stopped, questioned, or searched by police; ever abused or threatened by police; and ever experienced racial discrimination by the police or in the courts among New York City adults, 2017, by demographic characteristics

	Ever stopped, questioned, or searched by police			Ever abused or threatened by police			Ever experienced racial discrimination from the police or in the courts		
	Prevalence	Upper, lower 95% confidence intervals	*p*-value	Prevalence	Upper, lower 95% confidence intervals	*p*-value	Prevalence	Upper, lower 95% confidence intervals	*p*-value
**Sex**									
Male	43.3	39.0, 47.6	< .001	13.5	10.8, 16.8	< .001	17.8	14.7, 21.4	0.004
Female	17.2	14.7, 20.2	Ref	4.6	3.1, 6.7	Ref	11.7	9.4, 14.4	Ref
**Sexual identity**									
Gay/lesbian/bisexual	40.5	31.4, 50.3	0.032	5.2^*^	2.1, 12.1	0.118	10.6	6.2, 17.6	0.160
Heterosexual	29.6	26.8, 32.5	Ref	9.1	7.4, 11.3	Ref	15.0	12.8, 17.4	Ref
**Race/ethnicity**									
White, non-Latino	31.2	27.1, 35.6	Ref	5.2	3.5, 7.5	Ref	5.4	3.4, 8.3	Ref
Black, non-Latino	33.4	27.9, 39.4	0.546	16.3	11.7, 22.2	< .001	30.9	25.2, 37.2	< .001
Latino	29.7	25.1, 34.8	0.657	8.7	5.9, 12.6	0.072	16.2	12.5, 20.7	< .001
Asian/PI, non-Latino	14.9	10.5, 20.7	< .001	3.8^*^	1.7, 8.2	0.441	9.5	5.9, 15.0	0.109
Other or multi-races	34.2^*^	22.2, 48.6	0.677	30.8^*^	21.5, 41.9	< .001	24.3^*^	13.5, 39.7	0.006
**Birthplace**									
United States	37.5	34.0, 41.1	Ref	10.4	8.2, 13.1	Ref	16.7	13.9, 19.9	Ref
Outside United States	18.4	15.2, 22.1	< .001	6.8	4.7, 9.6	0.039	11.7	9.2, 14.8	0.018
**Age**									
18–24	31.1	23.3, 40.2	Ref	8.7	5.3, 14.1	Ref	12.0	7.9, 17.9	Ref
25–44	37.6	33.1, 42.5	0.187	12.4	9.2, 16.4	0.204	18.0	14.3, 22.3	0.066
45–65	27.4	23.3, 31.9	0.449	7.2	5.2, 9.8	0.522	15.7	12.6, 19.5	0.224
65 +	11.7	9.0, 15.1	< .001	3.0	1.7, 5.1	0.014	6.9	4.2, 11.1	0.092
**Household income**									
< 200% FPL	25.5	21.3, 30.3	0.005	11.7	8.5, 15.8	0.029	19.2	15.2, 23.9	0.009
≥ 200% FPL	33.9	30.3, 37.7	Ref	7.1	5.4, 9.2	Ref	12.6	10.4, 15.2	Ref
**Education**									
Less than HS graduate	28.1	21.1.36.4	0.342	14.1	8.9, 21.4	0.003	21.0	14.6, 29.3	0.009
HS graduate	26.5	21.1, 32.7	0.099	10.7	7.3, 15.3	0.005	17.6	13.0, 23.4	0.016
Some college	33.9	28.3, 39.9	0.653	10.4	6.9, 15.4	0.001	15.3	11.6, 20.0	0.057
College graduate	32.3	28.7, 36.2	Ref	4.6	3.3, 6.3	Ref	10.7	8.5, 13.2	Ref
**Employment status**									
Employed	31.2	27.8, 34.8	Ref	7.4	5.8, 9.5	Ref	13.6	11.1, 16.7	Ref
Unemployed	37.4	29.7, 45.8	0.166	11.0	6.4, 18.2	0.246	20.5	13.6, 29.8	0.115
Not in labor force	24.3	19.0, 30.6	0.047	12.0	7.6, 18.4	0.111	17.2	12.3, 23.6	0.262

Source: 2017 NYC Social Determinants of Health Survey Data were weighted to the adult residential population per the American Community Survey 2016 and age adjusted to the US 2000 standard population, except for age-specific estimates. *Estimate should be interpreted with caution. Estimate’s relative standard error (a measure of estimate precision) is greater than 30%, the 95% confidence interval (CI) half-width is greater than 10, or the sample size is too small, making the estimate potentially unreliable.95% CIs are a measure of estimate imprecision: the wider the CI, the more imprecise the estimate

**Table 2:** Prevalence of health outcomes and behaviors by ever being stopped, questioned, and searched by police; ever being threatened or abused by police; and experienced racial discrimination by the police or in the courts among New York City adults, 2017

	Prevalence	Upper, lower 95% confidence intervals	Prevalence	Upper, lower 95% confidence intervals	*p*-value
	Ever stopped, questioned, or searched by police		Never stopped, questioned, or searched by police		
Poor physical health in past 30 days	17.5	13.7, 22.2	11.0	9.2, 13.0	0.006
Poor mental health in past 30 days	20.4	16.2, 25.3	11.9	9.8, 14.5	0.001
Serious psychological distress	7.1	5.0, 10.0	6.0	4.6, 7.8	0.436
Binge drinking in past 30 days	27.4	23.0, 32.3	17.9	15.4, 20.7	< .001
	Ever threatened or abused by police		Never threatened or abused by police		
Poor physical health in past 30 days	29.4	21.1, 39.4	11.8	10.1, 13.7	< .001
Poor mental health in past 30 days	26.7	18.7, 36.5	13.5	11.5, 15.9	0.005
Serious psychological distress	13.8	8.4, 21.8	5.8	4.6, 7.3	0.021
Binge drinking in past 30 days	24.0	17.0, 32.7	20.7	18.3, 23.2	0.433
	Ever experienced racial discrimination from the police in the courts		Never experienced racial discrimination from the police or in the courts		
Poor physical health in past 30 days	17.7	12.5, 24.5	12.4	10.5, 14.4	0.095
Poor mental health in past 30 days	21.3	15.5, 28.4	13.3	11.2, 15.7	0.021
Serious psychological distress	8.6	5.5, 13.2	6.2	4.9, 7.9	0.249
Binge drinking in past 30 days	25.1	19.2, 32.2	20.1	17.8, 22.6	0.155

Source: 2017 Social Determinants of Health Survey

Data were weighted to the adult residential population per the American Community Survey 2016 and age adjusted to the US 2000 standard population

95% confidence intervals (CIs) are a measure of estimate imprecision: the wider the CI, the more imprecise the estimate

After adjusting for education, household income and US nativity, ever having been stopped by police (RR: 1.68; 95% CI 1.23, 2.30), ever having been abused by police (RR: 1.67; 95% CI 1.07, 2.60), and ever having experienced discrimination by police or in courts (RR: 1.52; 95% CI 1.02, 2.27) were all positively associated with poor mental health (Table 3). Having been stopped by police and having experienced police abuse was positively associated with poor physical health (RR: 1.74; 95%CI 1.27, 2.38 and RR: 2.27; 95% CI 1.51, 3.40 respectively), and police abuse was positively associated with serious psychological distress (RR: 2.27; 95% CI 1.31, 3.92). Having ever been stopped by police and having ever experienced discrimination by police or in courts was positively associated with binge drinking (RR: 1.47; 95% CI 1.16, 1.86 and RR: 1.39; 95% CI 1.05, 1.85, respectively).

**Table 3:** Unadjusted and adjusted relative risk estimates and their 95% confidence intervals (CI) for the association between health outcomes and ever being stopped, questioned, or search by police; ever being abused or threatened by police; and ever experienced racial discrimination by the police or in the courts among New York City adults, 2017

	Poor physical health in past 30 days			Poor mental health in past 30 days			Serious psychological distress			Binge drinking in past 30 days		
	RR	CI	*p*-value	RR	CI	*p*-value	RR	CI	*p*-value	RR	CI	*p*-value
**Unadjusted relative risks**												
Ever vs. never stopped	1.45	1.05, 1.99	0.026	1.73	1.28, 2.35	0.001	1.20	0.76, 1.89	0.432	1.67	1.34, 2.10	< .001
Ever vs. never abused	2.34	1.55, 3.55	< .001	1.90	1.25, 2.88	0.005	2.38	1.35, 4.19	0.004	1.22	0.85, 1.75	0.283
Ever vs. never discrimination	1.57	1.05, 2.35	0.032	1.68	1.17, 2.42	0.007	1.44	0.85, 2.43	0.176	1.30	0.96, 1.76	0.095
**Adjusted relative risks**												
Ever vs. never stopped	1.74	1.27, 2.38	0.002	1.68	1.23, 2.30	0.002	1.40	0.89, 2.21	0.150	1.47	1.16, 1.86	0.002
Ever vs. never abused	2.27	1.51, 3.40	< .001	1.67	1.07, 2.60	0.031	2.27	1.31, 3.92	0.005	1.27	0.90, 1.79	0.184
Ever vs. never discrimination	1.48	0.98, 2.24	0.069	1.52	1.02, 2.27	0.046	1.22	0.71, 2.10	0.485	1.39	1.05, 1.85	0.029

Source: 2017 NYC Social Determinants of Health Survey

Data were weighted to the adult residential population per the American Community Survey 2016

95% confidence intervals (CIs) are a measure of estimate imprecision: the wider the CI, the more imprecise the estimate

^*^Models were adjusted for education, household income, and U.S. nativity

The associations between having ever been stopped by police, abused by police, or having experienced discrimination by police or in courts and health outcomes varied by race and ethnicity (Table 4). Associations were more often observed among Black NYC residents, with the likelihood of reporting adverse physical, mental, or behavioral health outcomes up to three and a half times greater among people having ever been stopped by police, or having ever experienced police abuse or discrimination by police or in courts. Fewer associations were observed among White, Latino, and Asian/Pacific Islander residents as well as among those of other or multiple races. While some point estimates in these strata were of larger magnitude, their wide confidence intervals suggest they are possibly unreliable owing to smaller cell sizes.

**Table 4:** Adjusted* relative risk estimates of the association between health outcomes and ever being stopped, questioned, or searched by police; ever being abused or threatened by police; and ever experienced racial discrimination by the police or in the courts among New York City adults, 2017, by race/ethnicity

	Poor physical health in past 30 days			Poor mental health in past 30 days			Serious psychological distress			Binge drinking in past 30 days		
	RR	CI	*p*-value	RR	CI	*p*-value	RR	CI	*p*-value	RR	CI	*p*-value
**White, non-Hispanic**												
Ever vs. never stopped	1.49	0.91, 2.44	0.123	1.68	1.01, 2.80	0.055	1.50	0.70. 3.25	0.306	1.51	1.09, 2.07	0.014
Ever vs. never abused	1.59	0.70, 3.59	0.286	2.28	1.20, 4.36	0.021	4.66	2.28, 9.55	< .001	1.54	0.99, 2.41	0.086
Ever vs. never discrimination	0.72	0.23, 2.30	0.575	1.43	0.57, 3.59	0.462	1.37	0.40, 4.68	0.615	1.35	0.74, 2.44	0.368
**Black, non-Hispanic**												
Ever vs. never stopped	2.54	1.20, 5.38	0.023	1.72	0.84, 3.53	0.143	3.48	1.24, 9.71	0.027	1.25	0.68, 2.31	0.464
Ever vs. never abused	3.12	1.39, 7.02	0.012	1.36	0.49, 3.81	0.562	1.36	0.49, 3.81	0.562	0.72	0.27, 1,97	0.518
Ever vs. never discrimination	1.35	0.55, 3.32	0.513	1.17	0.52, 2.62	0.704	3.47	1.28, 9.41	0.021	1.86	1.05, 3.29	0.036
**Latino**												
Ever vs. never stopped	1.20	0.68, 2.11	0.535	1.67	0.95, 2.95	0.078	0.60	0.26, 1.39	0.237	1.80	1.12, 2.90	0.019
Ever vs. never abused	1.79	0.79, 4.05	0.198	1.65	0.78, 3.50	0.218	1.00	0.29, 3.39	0.996	1.28	0.65, 2.52	0.484
Ever vs. never discrimination	1.73	0.94, 3.18	0.094	1.78	0.95, 3.33	0.082	0.70	0.26, 1.89	0.482	1.06	0.60, 1.88	0.840
**Asian/PI, non-Latino**												
Ever vs. never stopped	1.51	0.69, 3.32	0.303	1.97	0.89, 4.35	0.108	2.52	0.64, 9.88	0.180	0.84	0.38, 1.85	0.588
Ever vs. never abused	3.39	1.39, 8.26	0.009	2.65	1.07, 6.52	0.053	1.26	0.09, 16.86	0.864	2.28	0.99, 5.28	0.073
Ever vs. never discrimination	3.02	1.43, 6.39	0.005	2.65	1.07, 6.52	0.053	1.30	0.21, 7.97	0.775	2.03	1.07, 3.85	0.018
**Other or multi-races**												
Ever vs. never stopped	6.74	2.11, 21.47	0.000	0.83	0.28, 2.50	0.655	4.41	1.04, 18.72	0.044	0.68	0.30, 1.53	0.340
Ever vs. never abused	5.78	1.90, 17.60	0.001	1.24	0.34, 4.43	0.662	2.35	0.40, 13.89	0.354	0.57	0.16, 2.10	0.359
Ever vs. never discrimination	4.48	1.26, 15.98	0.006	0.54	0.14, 2.00	0.180	0.82	0.24, 2.81	0.641	0.96	0.40, 2.33	0.935

Source: 2017 NYC Social Determinants of Health Survey

Data were weighted to the adult residential population per the American Community Survey 2016

95% confidence intervals (CIs) are a measure of estimate imprecision: the wider the CI, the more imprecise the estimate

^*^Models were adjusted for education, household income, and U.S. nativity

Variation was also observed across age groups (Table 5). Associations were most often observed among NYC adults aged 25–44 years, with people having ever been stopped by police, or having ever experienced police abuse or discrimination by police or in courts up to four times as likely to report adverse physical, mental, and behavioral health outcomes. Fewer associations were observed among the youngest (18–24 years), middle-aged (45–64 years), and oldest (65 + years) NYC residents, though the magnitude of some point estimates was similar to those of New Yorkers aged 25–44 years.

**Table 5:** Adjusted* relative risk estimates of the association between health outcomes and ever being stopped, questioned, or searched by police; ever being abused or threatened by police; and ever experienced racial discrimination by the police or in the courts among New York City adults, 2017, by age

	Poor physical health in past 30 days			Poor mental health in past 30 days			Serious psychological distress			Binge drinking in past 30 days		
	RR	CI	*p*-value	RR	CI	*p*-value	RR	CI	*p*-value	RR	CI	*p*-value
**Age 18–24**												
Ever vs. never stopped	1.38	0.46, 4.17	0.121	1.21	0.44, 3.33	0.705	1.79	0.40, 8.01	0.455	1.16	0.58, 2.29	0.677
Ever vs. never abused	0.96	0.21, 4.27	0.867	0.68	0.11, 4.32	0.671	6.95	3.19, 15.12	< .001	1.42	0.60, 3.34	0.447
Ever vs. never discrimination	2.33	1.33, 4.07	< .001	0.70	0.18, 2.80	0.609	2.25	0.64, 7.88	0.201	2.33	0.70, 2.84	0.336
**Age 25–44**												
Ever vs. never stopped	3.14	1.78, 5.56	< .001	1.69	1.04, 2.75	0.035	1.72	0.82, 3.61	0.155	1.43	1.06, 1.93	0.022
Ever vs. never abused	4.17	2.32. 7.49	< .001	1.57	0.82, 3.04	0.193	3.27	1.46, 7.33	0.006	1.14	0.75, 1.75	0.553
Ever vs. never discrimination	3.42	1.86, 6.30	< .001	1.40	0.74, 2.65	0.312	1.45	0.60, 3.52	0.413	0.94	0.61, 1.46	0.795
**Age 45–65**												
Ever vs. never stopped	1.38	0.88, 2.15	0.168	1.67	1.02, 2.74	0.043	0.97	0.46, 2.04	0.933	1.32	0.83, 2.09	0.246
Ever vs. never abused	1.80	1.04, 3.14	0.054	1.89	1.05, 3.39	0.045	1.93	0.84, 4.43	0.139	1.93	0.84, 4.43	0.139
Ever vs. never discrimination	0.91	0.50, 1.67	0.770	1.62	0.95, 2.77	0.084	0.89	0.39, 2.03	0.783	2.34	1.50, 3.67	0.001
**Age 65 < **												
Ever vs. never stopped	1.56	0.89, 2.75	0.155	1.85	0.75, 4.58	0.211	2.06	0.67, 6.31	0.234	1.68	0.77, 3.65	0.207
Ever vs. never abused	2.42	1.41, 4.16	0.010	3.45	1.55, 7.67	0.013	5.12	2.12, 12.35	0.005	1.36	0.68, 2.71	0.406
Ever vs. never discrimination	0.49	0.15, 1.54	0.196	1.02	0.35, 3.03	0.966	0.97	0.22, 4.22	0.966	0.38	0.05, 2.67	0.315

Source: 2017 NYC Social Determinants of Health Survey

Data were weighted to the adult residential population per the American Community Survey 2016

95% confidence intervals (CIs) are a measure of estimate imprecision: the wider the CI, the more imprecise the estimate

^*^Models were adjusted for education, household income, and U.S. nativity

## Discussion

The criminal legal system is one of multiple systems of oppression that disproportionately impacts and marginalizes Black, Latino, and sexual minority individuals by exposing them to adverse conditions such as aggressive policing practices [7, 8, 22, 34, 35]. We examined the associations of policing practices and discrimination with health among a representative sample of adult NYC residents, as well as whether those associations varied by race and ethnicity and age. We found that approximately one-third (29%) had ever been stopped by the police, close to 10% had ever been physically abused by police and almost 15% had ever experienced racial discrimination from the police or in the courts. We also found that gay, lesbian, and bisexual New Yorkers were more likely to report being stopped by police than heterosexual New Yorkers and Black New Yorkers were more likely to report being abused by police and experience racial discrimination by the police or in courts than White New Yorkers. Our findings on rates of stops and abuse contribute to a growing body of literature on the quantity and character of police contacts in large urban areas. Analyses by Geller and colleagues focusing on young adult men in NYC [25] found a much higher rate of stops (85%), while Hirschtick and colleagues found that 21% of residents of select Chicago neighborhoods experienced at least three police stops and 4% experienced stops characterized as aggressive [36]. Notably, we did not find differences across race and ethnicity in stops, although Black and Latino New Yorkers were disproportionately stopped based on stop, question, and frisk data [10]. However, our assessment of police stops had a lifetime timeframe and was not specific to street stops, a focus of aggressive policing practices. We did find, however, that Black New Yorkers were more likely to report having ever been abused by the police and that Black and Latino New Yorkers were more likely to report having ever experienced racial discrimination by police or in courts than White New Yorkers. These latter findings reflect racial and ethnic differences in interpersonal experiences with the police.

We also found that NYC residents with the lived experiences of police contact and discrimination were more likely to report several poor health outcomes relative to those who had not had those experiences with police. Having been stopped by the police was associated with poor physical and mental health and with binge drinking; having been abused by the police was associated with poor physical and mental health and with psychological distress; and having experienced racial discrimination by police and the courts were associated with poor mental health and binge drinking. Informed by the ecosocial theory of disease distribution, we also examined whether associations between police contacts and discrimination by the police or in court and health outcomes varied by race and ethnicity and age. We hypothesized that the associations between police contact and racial discrimination by the police and courts and health outcomes would be stronger among individuals socially marginalized because of race and ethnicity and older age groups. We found that the associations were more common and most consistently stronger among Black New Yorkers than for other racial and ethnic groups. Our findings on older New Yorkers were not consistent with our study hypothesis, as the association between having ever been stopped or abused by police or having ever faced discrimination by police or in courts with poor health outcomes was most often observed among New Yorkers aged 25 to 44 years as compared to their younger and older counterparts. While all New Yorkers have resided in a city with aggressive policing practices, the 25- to 44-year-old cohort would have been exposed on an ongoing basis to these practices which initiated during their young adult years, which may explain our findings among this age group.

Our finding that NYC residents who had ever had contact with the police were more likely to experience poor health outcomes is generally consistent with previous research [24, 26]. Similar to Geller et al. (2014), who found police contact associated with poor mental health among young adult men [25], we observed an association for serious psychological distress among younger adult New Yorkers who had ever been abused by police, but did not identify further associations between mental health measures and other experiences of police contact. Geller et al. (2014) examined associations between recent police contacts and health during the peak years of SQF (2012–2013), whereas our study asked about lifetime police contact [10]. We did not find the 2-way interaction term for sex significant in our analyses, and therefore, we did not stratify by sex, which may have obscured the experience of younger men in our analyses.

Our findings on Black New Yorkers are consistent with previous research indicating that police contacts may be harmful to the physical and mental health of Black individuals throughout the US [26, 34, 35, 37–39]. A systematic literature review identified, across studies, a nearly twofold higher prevalence of poor mental health among Black Americans who report any prior police interaction compared to those who did not [34]. In their study on multiple lifetime police interactions and mental health, Hirschtick et al. (2020) found that those who reported a higher number of lifetime police stops had three times greater odds of experiencing post-traumatic stress disorder (PTSD) symptoms compared to those who reported fewer stops [36]. These findings echo those of Geller et al. (2014) and Jackson et al. (2019), whose research indicated that more intrusive police contacts were associated with current and lifetime trauma, anxiety symptoms, and/or emotional distress [25, 38]. Additionally, Black residents in our study were more likely than their racial and ethnic counterparts to be subjected to abusive police behavior [40]. Together, these findings may indicate the particular salience for the health of any racialized exposures for Black Americans because they are reminders of other, past, and historical racial traumas [41] and that the effects of these exposures are unique and extend beyond the typical effects of other violence exposures on mental health [20].

A major strength of our study is the use of 2017 SDH Survey data, which included information on lifetime exposure of police contact and discrimination by police and the courts as well as several sociodemographic characteristics and health outcomes. This dataset allowed the opportunity to examine the relationship between these variables in a weighted sample that is representative of the adult NYC population.

Study limitations included the cross-sectional nature of the survey data which restricted our ability to establish the temporal relation of police contact and discrimination with poor health outcomes, as well as our ability to establish causality. As suggested by the increase in reports of anxiety and symptoms of depression among Black people observed in the wake of George Floyd’s murder [42, 43], the increasingly publicized nature of police violence experienced via the media may contribute to poor mental health [44]; this was not accounted for in our analysis. Despite a high cooperation rate among participants contacted, the low response rate among eligible participants may potentially hamper generalizability. Furthermore, the data did not include information on the timing and frequency of police contact or court experiences during the life course or on the geographic location of said experiences. While we acknowledge the concentration of policing in particular neighborhoods, as well as the role of the built environment in shaping health, the dataset did not contain elements that permit us to control for or explore geographic variability. Underreporting of police contact is possible given the sensitive nature of the topic and could be differential across racial and ethnic and age groups. Potential recall bias, especially among older adults, could have also resulted in underreporting of police contact, though the higher likelihood of recalling negative police contacts than positive ones may mean that our findings on the associations between police contacts and health may be biased towards contacts assessed as negative [45]. Past research [46, 47] has highlighted the disproportionate police contact and interaction with the carceral system experienced by people with darker complexions, and as such, the absence of data on colorism in our analysis is an important limitation. Our measures were self-reported with inherent limitations related to what respondents were willing to share and social desirability bias [48]. We did not adjust for multiple comparisons in our analysis; had we done so, it is possible that fewer findings would be statistically significant. Finally, we did not test measurement nonequivalence for the outcomes of interest, though past research has highlighted some estimation differences for Kessler-6 across racial/ethnic groups [49]. Notwithstanding limitations, our findings are relevant to aggressive policing practices employed in cities throughout the US and their association with adverse health outcomes.

### Conclusion

Public policies related to the criminal legal system in the US inequitably impact the health of socially marginalized communities. Policing policies are no exception. Many police departments in large urban areas have embraced aggressive policing practices such as SQF and quality-of-life policing with the goal of preventing disorder and deterring crime. Black and Latino New Yorkers have been inequitably subjected to decades of these aggressive policing practices, which we have found to be associated with a range of poor health outcomes. Our findings, in combination with the growing body of literature on the relationship between criminal legal exposures and health, can inform municipalities and states in the US that are increasingly considering changes to policing and criminal legal approaches [50–52], in particular community-based and health-centered approaches to community safety. These include efforts to shift more resources towards community services such as substance use disorder treatment and housing [53]; increasing transparency and accountability for police misconduct [54]; decriminalizing or de-prioritizing arrest for low-level crimes associated with substance use or poverty [55]; and programs to dispatch health professionals for mental health emergencies, rather than police [56]. Finally, as noted by the American Public Health Association [51], the epidemic of police violence is a public health crisis. It is urgent that public health surveillance systems include data on the impact of exposure to police contact and discrimination and to the carceral system on health outcomes. This would allow researchers to highlight the critical evidence to inform and reform policies that disproportionately impact individuals and communities of color.
